# RhoC is a major target of microRNA-93-5P in epithelial ovarian carcinoma tumorigenesis and progression

**DOI:** 10.1186/s12943-015-0304-6

**Published:** 2015-02-04

**Authors:** Xi Chen, Shuo Chen, Yin-Ling Xiu, Kai-Xuan Sun, Zhi-Hong Zong, Yang Zhao

**Affiliations:** Department of Gynecology, The First Affiliated Hospital of China Medical University, Shenyang, 110001 P. R. China; Department of Biochemistry and Molecular Biology, College of Basic Medicine, China Medical University, Shenyang, 110001 P. R. China

**Keywords:** MiR-93-5P, RhoC, Ovarian epithelial carcinoma, Tumorigenesis and progression

## Abstract

**Background:**

An increasing amount of evidence has revealed that microRNAs regulate various biological processes, including cell differentiation, cell proliferation, apoptosis, drug resistance, and fat metabolism. Studies have shown that miR-93’s targetome in cancer has not been fully defined. Moreover, the role of miR-93 in epithelial ovarian carcinoma (EOC) remains largely unknown.

**Methods:**

*MIR-93* mRNA expression in normal ovarian tissue, benign tumors, borderline tumors, primary ovarian carcinomas, and metastatic omentum was quantified. The ovarian carcinoma cell lines OVCAR3, SKOV3/DDP, and HO8910-PM were transfected with miR-93-5P, after which cell phenotype and expression of relevant molecules were assayed. Dual-luciferase reporter assay and a xenograft mouse model were used to examine miR-93 and its target gene *RHOC* (Ras homolog gene family member C).

**Results:**

*MIR-93* mRNA expression was significantly lower in ovarian carcinomas and borderline tumors than in normal ovarian tissues (p < 0.05), and was lower in metastatic omentum than in relative primary ovarian carcinomas (p < 0.05). *MIR-93* mRNA expression was also negatively associated with differentiation (well vs. poor and moderate) and International Federation of Gynecology and Obstetrics staging (FIGO stage I/II vs. stage III/IV) in ovarian carcinoma (p < 0.05), besides, miR-93 was higher expressed in mucinous adenocarcinoma than the other types (p < 0.05). MiR-93-5P overexpression reduced proliferation (p < 0.05); promoted G_1_ or S arrest and apoptosis (p < 0.05); suppressed migration and invasion (p < 0.05); and reduced RhoC, P70S6 kinase, Bcl-xL, matrix metalloproteinase 9 (MMP9) mRNA or protein expression; conversely, it induced P53 and cleaved PARP expression (p < 0.05). Dual-luciferase reporter assay indicated that miR-93 directly targeted RhoC by binding its 3′ untranslated region. MiR-93-5P transfection also suppressed tumor development and RhoC expression (determined by immunohistochemistry) *in vivo* in the xenograft mouse model (p < 0.05).

**Conclusions:**

This is the first demonstration that miR-93-5P may inhibit EOC tumorigenesis and progression by targeting *RhoC*. These findings indicate that miR-93-5P is a potential suppressor of ovarian cellular proliferation. The involvement of miR-93-5P–mediated RhoC downregulation in inhibiting EOC aggressiveness may provide extended insight into the molecular mechanisms underlying cancer aggressiveness.

## Background

Ovarian cancer is the most lethal of the gynecologic malignancies, but relatively little is known about the molecular genetics of its initiation and progression. The primary genetic alterations associated with epithelial ovarian cancer, which accounts for 90% of ovarian cancer, remain to be identified [1]. As recurrence and metastasis greatly affect the prognosis of ovarian cancer, the 5-year survival rate for all stages of ovarian cancer has been estimated to be 35–38%. Due to the high mortality of epithelial cancer, exploring the related molecular mechanisms of epithelial ovarian carcinoma (EOC) initiation and development, and identifying the major factors of invasion and metastasis would be of great significance for the treatment and prognosis of EOC.

Ras homolog gene family member C (RhoC) is a small G protein/guanosine triphosphatase involved in tumor mobility, invasion, and metastasis. Previously, we showed that RhoC expression correlated to clinical stage and vascularization in ovarian cancer [2]. Upregulated RhoC may affect ovarian carcinogenesis and should be considered a good biomarker of ovarian carcinoma differentiation and progression. RhoC plays an important role in apoptosis by modulating the relevant genes and phosphorylation of the downstream P70S6 kinase (P70S6K) [3]. Most studies show that RhoC has multiple functions in tumor metastasis, orchestrating the action of multiple downstream effectors, degradation, and reconstruction of the extracellular matrix.

MicroRNAs (miRNAs) are noncoding, single-stranded RNAs ~22 nucleotides in length that constitute a novel class of gene regulators. They regulate gene expression negatively at the post-transcriptional level by binding to imperfect complementary sites at the 3′ untranslated region (UTR) of their target mRNA transcripts [4]. The binding of miRNAs to their target mRNAs leads to translational repression or decreases the stability of the mRNA. MiRNAs regulate various biological processes, including cell differentiation, cell proliferation, apoptosis, drug resistance, and fat metabolism [5]. MiR-93, derived from a paralogue (miR-106b-25) of the miR-17-92 cluster [6-8], has been reported involved in colorectal, breast, pancreatic, and lung cancer and hepatocellular carcinoma. The identified targets of miR-93 include P21, cyclin B1 (*CCNB1*), ERBB2 [9], Akt3, SOX4, STAT3 [10], vascular endothelial growth factor A (*VEGFA*) [11], and Smad7 [12], suggesting that miR-93 may play tumor suppressor roles through diverse mechanisms. However, the targetome of miR-93 in EOC has not been fully defined so far. The seed region we predicted in the 3′ UTR of *RHOC* showed that *RHOC* is a direct target of miR-93. The involvement of RhoC and miR-93 in EOC remains unknown. This is the first study to determine the involvement of miR-93 and RhoC in the EOC.

## Results

### Correlation of *MIR93* mRNA expression with ovarian carcinoma pathogenesis and aggressiveness

**Figure 1:**
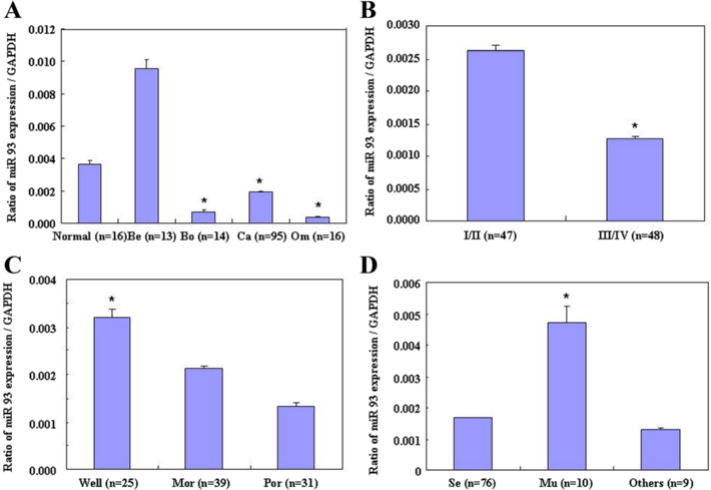
**Correlation of** ***MIR93*** **mRNA expression with ovarian carcinoma pathogenesis and aggressiveness.** *MIR93* mRNA expression was significantly lower in ovarian carcinomas and borderline tumors than in normal ovarian tissues, and lower in metastatic omentum than in relative primary ovarian carcinomas **(A)**, and was negatively associated with International Federation of Gynecology and Obstetrics (FIGO) staging (stage I/II vs. stage III/IV, **B)**, differentiation (well vs. poor and moderate, **C)** and pathological subtype (mucinous vs. the other types, **D)** in ovarian carcinoma. Be = Benign, Bo = Borderline, Om = Omentum, Mu = mucinous, Se = Serous.

MiR-93 was quantified in normal ovary tissue, benign and borderline tumors, and primary ovarian carcinoma using real-time PCR. *MIR93* mRNA expression was significantly lower in ovarian carcinomas and borderline tumors than in normal ovarian tissues, and lower in metastatic omentum than in relative primary ovarian carcinomas (Figure 1A, p < 0.05), and was negatively associated with International Federation of Gynecology and Obstetrics (FIGO) staging (stage I/II vs. stage III/IV, Figure 1B, p < 0.05), differentiation (well vs. poor and moderate, Figure 1C, p < 0.05) and pathological subtype (mucinous vs. the other types, Figure 1D, p < 0.05) in ovarian carcinoma. However, there were no significant differences in expression among ages (p > 0.05, data not shown).

### Effects of miR-93-5P transfection on ovarian carcinoma cell phenotype *in vitro*

**Figure 2:**
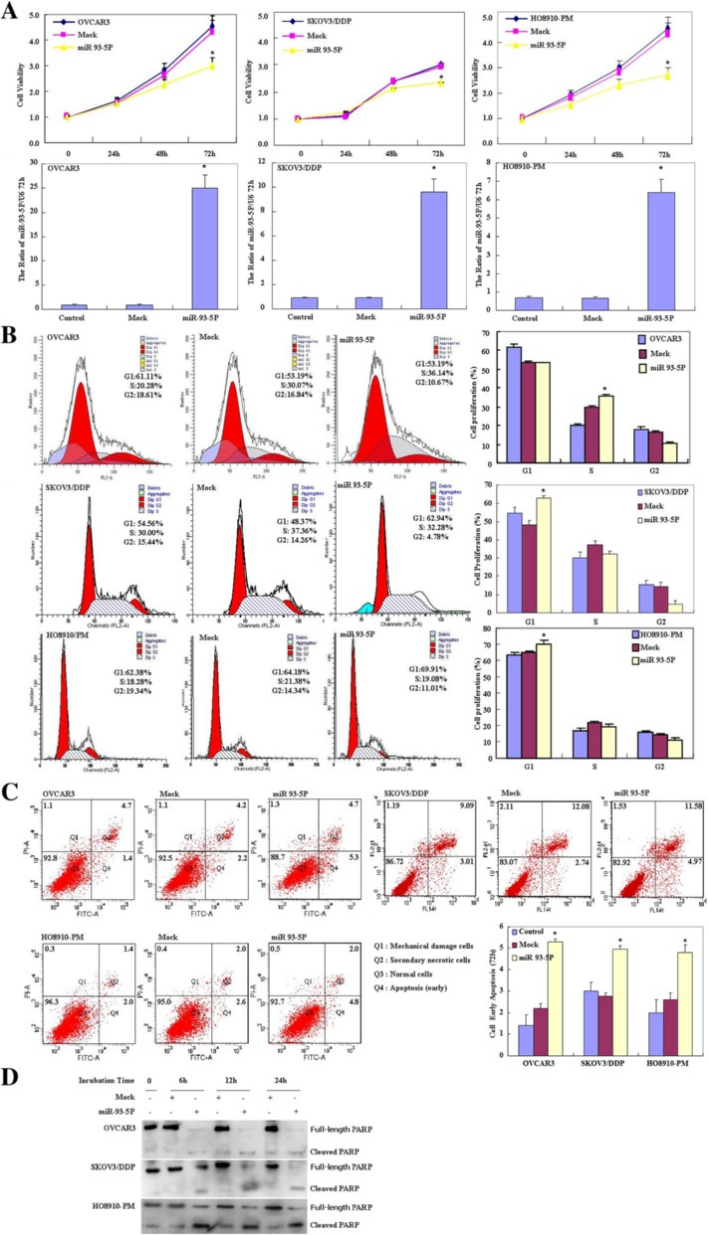
**Effects of miR-93-5P transfection on ovarian carcinoma cell proliferation, cell cycle and cell apoptosis of ovarian carcinoma cell lines in vitro.** Following miR-93-5P transfection, OVCAR3, HO8910-PM, SKOV3/DDP cell lines exhibited significantly slower growth and higher mature miR-93-5P mRNA expression **(A)**, induced G_1_ or S arrest **(B)**, early apoptosis **(C)** and cleaved PARP expression **(D)** than the control and mock-transfected cells. Results are representative of three separate experiments; data are expressed as the mean ± standard deviation, *P < 0.05.

**Figure 3:**
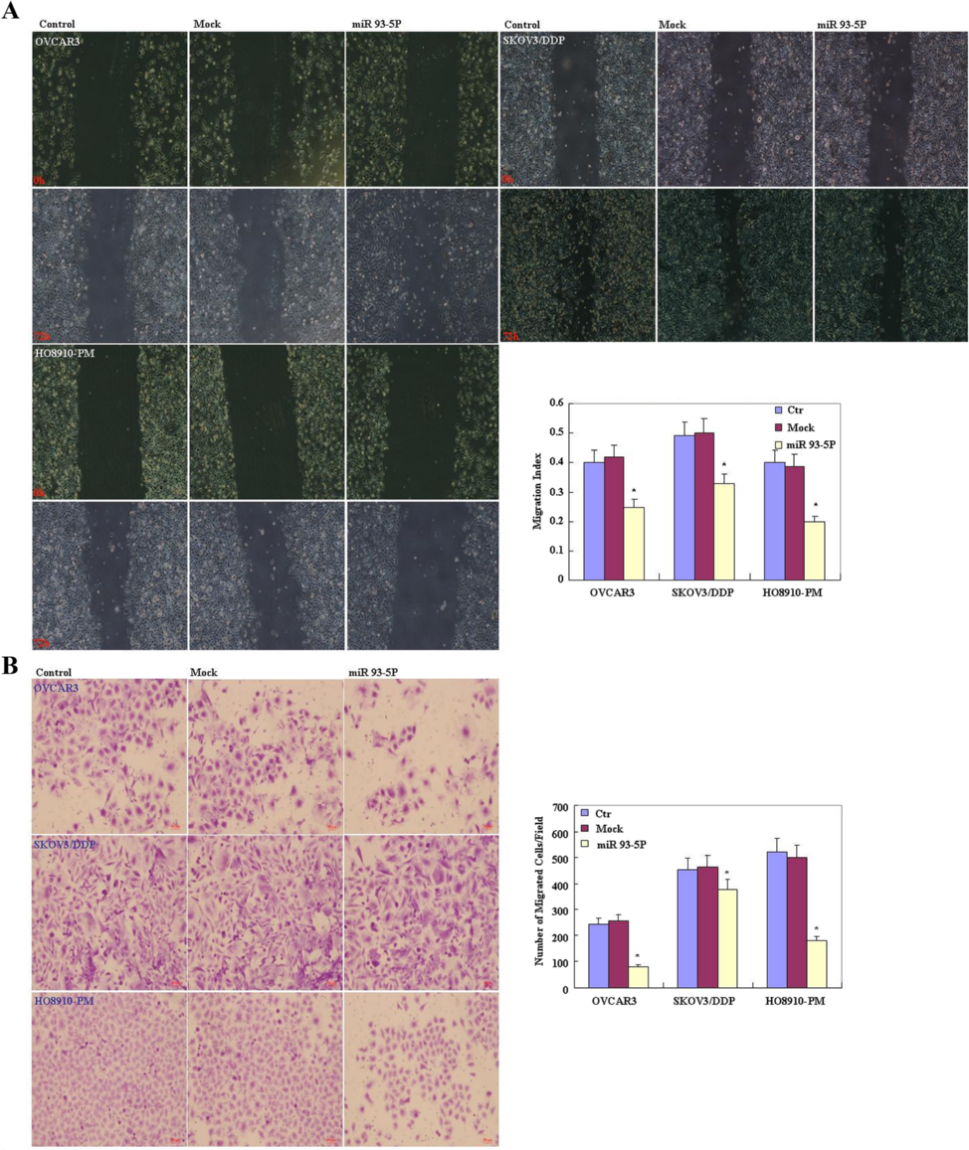
**Effects of miR-93-5P transfection on the invasive and metastatic ability of ovarian carcinoma cell lines.** After transfection with the miR-93-5P mimic, ovarian carcinoma cell lines showed lower migration in wound healing assays **(A)**, and slower invasion in matrigel transwell assays **(B)** compared with the control and mock cells. Results are representative of three separate experiments; data are expressed as the mean ± standard deviation, *P < 0.05.

OVCAR3, HO8910-PM, SKOV3/DDP cell lines were transfected with miR-93-5P. Following transfection, the cells exhibited significantly slower growth than the control and mock-transfected cells (Figure 2A, p < 0.05, Cell Counting Kit-8 [CCK-8] assay), and cells exhibited significantly up-regulated mature miR-93-5P mRNA expression (Figure 2A, p < 0.05). Propidium iodide (PI) staining and flow cytometry revealed significant induction of G_1_ or S arrest in miR-93-5P transfectants (Figure 2B, p < 0.05). MiR-93-5P transfection induced significantly higher levels of apoptosis (Figure 2C, p < 0.05, annexin V–fluorescein isothiocyanate [FITC] staining) and cleaved PARP expression (Figure 2D, p < 0.05), reduced cell migration (Figure 3A, p < 0.05, wound healing assay) and invasion (Figure 3B, p < 0.05, Transwell invasion assay) compared to control and mock-transfected cells.

### Effects of miR-93-5P transfection on ovarian carcinoma cell genotype *in vitro*

**Figure 4:**
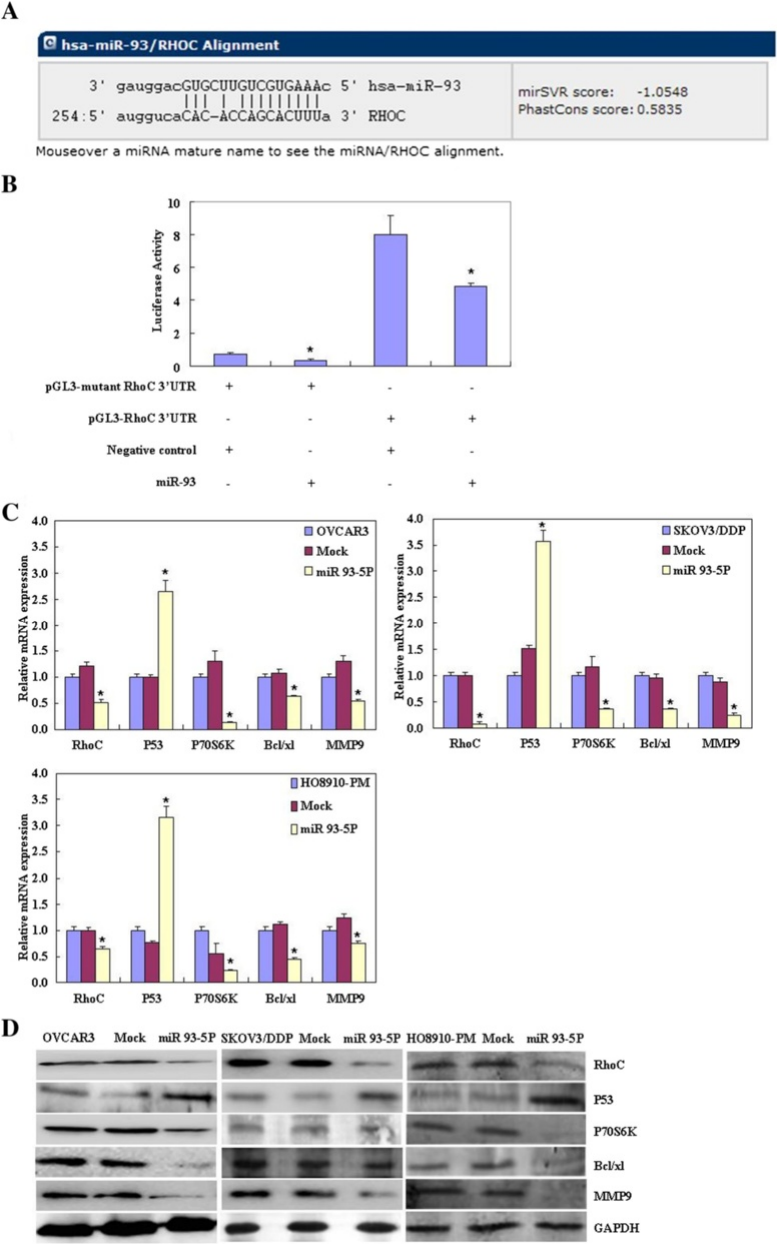
**Effects of miR-93**-**5P transfection on ovarian carcinoma cell genotype in vitro.** The predicted seed region in the 3′ UTR of *RHOC* showed that *RHOC* was a direct target of miR-93 **(A)**; dual-luciferase reporter assay indicated that miR-93 directly targeted *RHOC* by binding its 3′ UTR **(B)**. MiR-93-5P overexpression reduced RhoC, P70S6K, Bcl-xL, and MMP9 mRNA or protein expression while inducing P53 expression **(C & D)**.

The predicted seed region in the 3′ UTR of *RHOC* showed that *RHOC* was a direct target of miR-93 (Figure 4A); dual-luciferase reporter assay indicated that miR-93 directly targeted *RHOC* by binding its 3′ UTR (Figure 4B). Reverse transcription (RT)-PCR and western blotting showed that miR-93-5P overexpression reduced RhoC, P70S6K, Bcl-xL, and MMP9 mRNA or protein expression while inducing P53 expression (Figure 4C & D, p < 0.05).

### MiR-93-5P inhibited tumor growth *in vivo*

**Figure 5:**
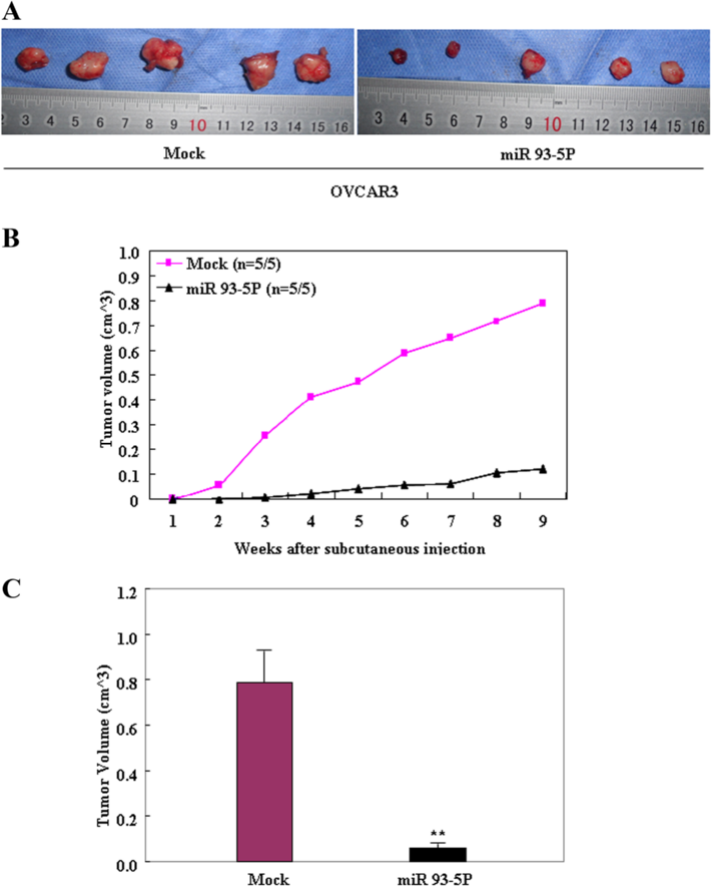
**MiR-93-5P inhibited tumor growth in vivo.** Tumor xenograft volume in nude mice treated with miR-93-5P was smaller than that in mock nude mice **(A)**. Tumor xenograft growth in miR-93-5P–treated nude mice was slower than that in the control group from day 4 and week 2 onwards **(B)**, and the DV increased in the latter period **(C)**.

Tumor xenograft volume in nude mice treated with miR-93-5P was smaller than that in mock nude mice (Figure 5A, p < 0.05). Tumor xenograft growth in miR-93-5P–treated nude mice was slower than that in the mock group from day 4 and week 2 onwards (Figure 5B, p_day 4_ < 0.05; p_deviation of tumor xenograft volume [DV]_ < 0.01, and p_week 2_ < 0.05; p_DV_ < 0.01), and the DV increased in the latter period (Figure 5C).

### MiR-93-5P downregulated RhoC expression in tumor xenografts *in vivo*

**Figure 6:**
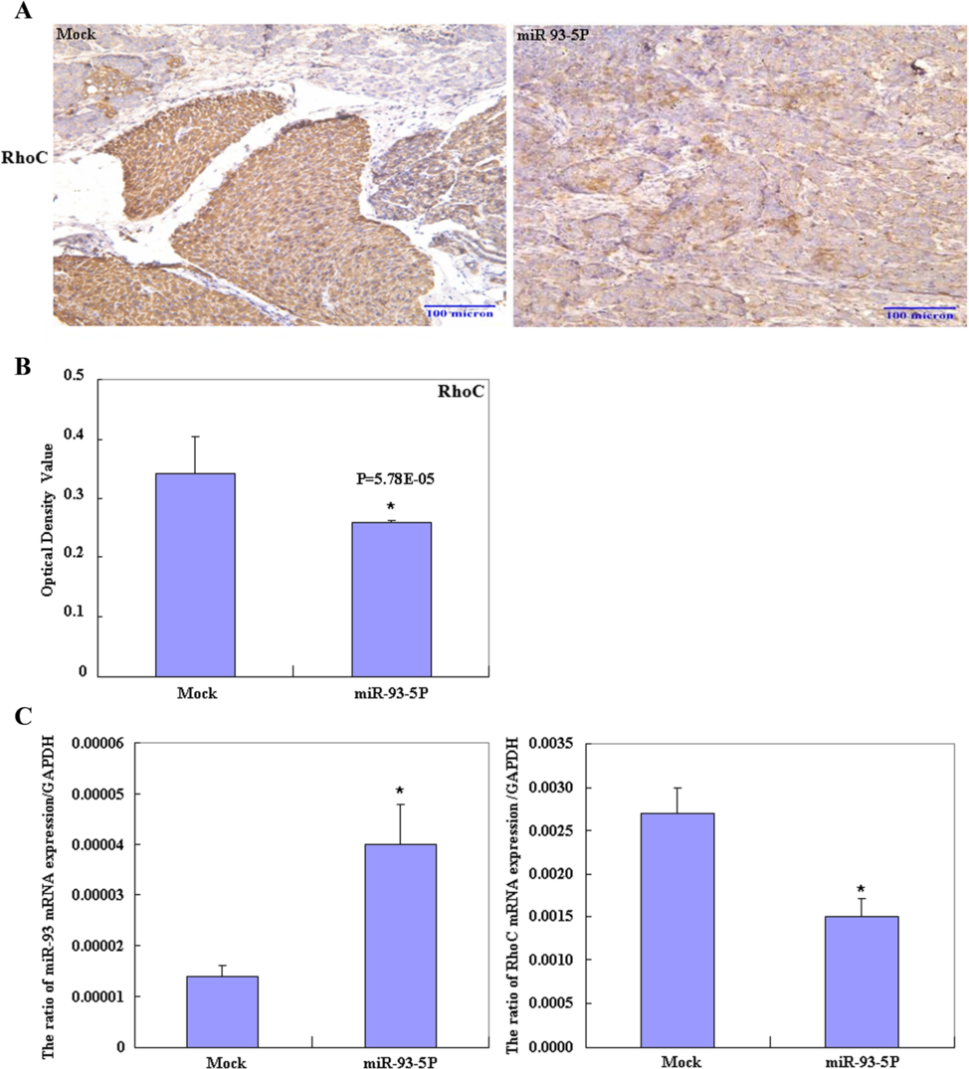
**MiR-93-5P downregulated RhoC expression in tumor xenografts in vivo.** IHC **(A)** and Optical Density Value **(B)** indicated that RhoC expression in the tumor xenografts of miR-93-5P–treated nude mice were decreased compared with that in the mock nude mice. miR-93-5P–treated nude mice showed induced miR-93 mRNA expression while suppressed RhoC mRNA expression **(C)**.

Immunohistochemistry (IHC) and Optical Density Value indicated that RhoC expression in the tumor xenografts of miR-93-5P–treated nude mice were decreased compared with that in mock nude mice (Figure 6A & B). Besides, miR-93-5P–treated nude mice showed induced miR-93 mRNA expression while suppressed RhoC mRNA expression (Figure 6C).

## Discussion

Given that many studies currently focus more on the tumor development mechanisms and effective cancer treatments, miRNAs have turned out to be a promising avenue of research. MiRNAs contribute to the regulation of their target genes by affecting base pairing to the 3′ UTR of a target mRNA, which results in either mRNA degradation or translation inhibition [13,14]. The difference in miRNA expression may contribute to tumor development [15-19]. An increasing number of studies have revealed that miRNAs are promising diagnostic and prognostic molecular biomarkers as well as therapeutic targets in cancer [20,21]. MiR-93, a typical member of the miR-106b-25 cluster, is involved in many cellular biological processes. Tang *et al*. [12] demonstrated that miR-93 suppresses colorectal cancer development via downregulating Wnt/β-catenin by targeting Smad7. Recently, Long *et al*. [11] also reported that miR-93 has a modulatory effect on VEGF expression and its downstream signaling, which might play important roles in the pathogenesis of diabetic nephropathy. Our results show that *MIR93* mRNA expression was significantly lower in ovarian carcinomas, borderline tumors, and metastatic omentum, and was negatively associated with differentiation and FIGO staging in ovarian carcinoma. These findings indicate that miR-93 might affect ovarian epithelial carcinogenesis and subsequent progression.

RhoC overexpression has been detected in many cancers such as gastric and breast cancer [22,23]. RhoC upregulation has been closely linked to growth, metastasis, and invasion in various malignancies [24-26]. Our previous works showed that RhoC overexpression promote ovarian epithelial carcinoma’s progression, and adverse prognosis. The seed region we predicted in the 3′ UTR of *RHOC* showed that *RHOC* is a direct target of miR-93. In addition, the dual-luciferase reporter assay indicated that following co-transfection with miR-93-5P and RhoC (wild type, Wt), the relative firefly luciferase activity, normalized with *Renilla* luciferase, was significantly lower than that in the RhoC group while being higher than that in the miR-93-5P group. Therefore, we performed further studies to explore the involvement of RhoC and miR-93-5P in ovarian cancer.

We found that after miR-93-5P transfection, ovarian cancer cells exhibited significantly slower growth than control and mock-transfected cells and significantly induced G_1_ (SKOV3/DDP and HO8910-PM) or S (OVCAR3) arrest and apoptosis, besides, miR-93-5P transfection also up-regulated cleaved PARP expression level, indicating that miR-93-5P might inhibit ovarian carcinoma cell proliferation and induced cell apoptosis. Additionally, miR-93-5P transfection decreased P70S6K and Bcl-xL expression while increasing P53 expression at both mRNA and protein level. P70S6K is one of the downstream effectors of the PI3K/AKT signal transduction pathway; it phosphorylates the S6 protein of the 40S ribosomal subunit and thus plays a role in protein synthesis and cell growth. Studies have shown that the P14ARF/MDM2/P53 pathway (P53 pathway) and the P70S6K/P85S6K pathway function at the G_1_/S checkpoint, suggesting that miR-93 might reduce cell proliferation, inducing G_1_ or S arrest by targeting P70S6K and P53 while repressing apoptosis by downregulating the transcription and translation of the anti-apoptotic Bcl-xL gene (*BCL2L1*). Furthermore, miR-93 overexpression reduced RhoC, and MMP9 mRNA or protein expression. According to the literature, these molecules are involved in cancer cell regulation, invasion, and metastasis [27-33]. Previously, we showed that RhoC promoted ovarian cancer invasion and metastasis through MMP9. We also found that RhoC knockdown was followed by reduced mRNA or protein expression of Bcl-xL, and phosphorylated P70S6K. Taken together, these results suggest that miR-93-5P may inhibit cell proliferation, resistance to apoptosis, and invasion by modulating the expression of these genes by targeting RhoC.

Our subsequent *in vivo* tumor xenograft studies showed that miR-93-5P inhibited EOC tumor growth. IHC staining and OD value indicated that RhoC expression in the tumor xenografts of miR-93-5P–treated nude mice were decreased, and miR-93-5P trasnsfection induced miR-93 mRNA expression while suppressed RhoC mRNA expression *in vivo*. These results indicate that miR-93-5P might suppress tumor proliferation and metastasis by targeting RhoC.

In conclusion, this is the first demonstration that miR-93-5P may inhibit EOC tumorigenesis and progression by targeting RhoC. These findings indicate that miR-93-5P is a potential suppressor of ovarian cellular proliferation. The involvement of miR-93-5P–mediated RhoC downregulation in inhibiting EOC aggressiveness may yield further insight into the molecular mechanisms underlying cancer aggressiveness.

## Materials and methods

### Cell culture and transfection

Ovarian carcinoma cell lines OVCAR3 (serous cystic adenocarcinoma), HO8910-PM (highly invasive HO8910, serous cystic adenocarcinoma) were obtained from the American Type Culture Collection (ATCC), SKOV3/DDP (cisplatin-resistant SKOV3, serous papillary cystadenocarcinoma) was purchased from Tumor Cell Bank of the Chinese Academy of Medical Science (Peking, China). They were cultured in RPMI 1640 (HyClone, Logan, UT, USA) supplemented with 10% fetal-bovine serum (FBS), 100 U/mL penicillin, and 100 μg/mL streptomycin. They were maintained in an incubator at 37°C under a humidified atmosphere of 5% CO2. The medium was changed every two or three days according to the recommended culture condition. Cell transfection was performed using lipofectamin 2000 ragent following the manufactures’ protocol. The miR-93-5P homologous target sequence was 5′-CAA AGU GCU GUU CGU GCA GGU AG-3′.

### Cell viability assay

Cell proliferation affected by miR-93-5P was measured using CCK-8 (Dojindo, Tokyo, Japan) method. Briefly, 2 × 103 cells were seeded onto each well of 96-well plate in 100 μL medium and incubated until adhesion. Another 24 hours were needed for starvation medium incubation. Cells were then challenged with 50 nM/L miR-93-5P mimic or mock mimic. At the time point of 0 h, 12 h, 24 h, 48 h and 72 h, 10 μL CCK-8 solutions was added to each well and incubated for 4 hours. The absorbance values at 450 nm was measured using a microplate reader.

### Cell cycle analysis

After incubation at 37°C in 5% CO_2_, the cells were trypsinized, harvested, washed twice in PBS, and fixed in 10 mL ice-cold ethanol for at least 2 h. The cells were then washed twice in PBS and incubated with 500 μL RNase (0.25 mg/mL) at 37°C for 30 min. Cells were pelleted by centrifugation, resuspended in 50 μg/mL PI (KeyGen, Nanjing, China), and incubated at 4°C in the dark for 30 min. The PI signal was examined using flow cytometry.

### Apoptosis assay

Flow cytometry was performed using cells stained with PI and FITC-labeled annexin V (KeyGen) to detect phosphatidylserine externalization as an endpoint indicator of early apoptosis, as described in the manufacturer’s instructions. Briefly, after 72-h incubation at 37°C in 5% CO_2_, cells were washed twice with cold PBS, resuspended in 1× binding buffer at 1 × 10^6^ cells/mL, and incubated with 200 μL binding buffer and 10 μL annexin V–FITC. The samples were gently vortexed and incubated for 15 min at 25°C in the dark before 300 μL binding buffer and 5 μL PI were added to each tube, and flow cytometry was performed within 1 h.

### Wound healing assay

Cells were seeded in 6-well culture plates for confluence; the confluent monolayer was scratched with a pipette tip, washed with PBS, and cultured in FBS-free medium. Cells were photographed at 0, 12, 24, 48 and 72 h; the wound area was measured using Image J software (National Institutes of Health, Bethesda, MD, USA). The wound healing rate = (Area of original wound − Area of actual wound at different times)/Area of original wound × 100%.

### Cell invasion assay

Matrigel (40 μL from 8 mg/mL stock solution; Becton-Dickinson Labware, Bedford, MA, USA) was overlaid on the upper surface of 6.5-mm Transwell chambers (8-μm pore size; BD Bioscience, San Jose, CA, USA). After incubating at 37°C for 4 h, culture medium was added to the bottom Transwell chamber. Cells were resuspended in serum-free RPMI 1640 medium, and 5 × 10^4^ cells were added to the top Transwell chambers. Following 72-h incubation, cells that had not invaded through the Matrigel were removed from the upper surface using cotton swabs. Cells invaded through the Matrigel and reached the bottom surface of the filters were fixed in methanol and stained with 0.1% crystal violet. Invasion was quantified by counting the number of cells under an Olympus fluorescence microscope (Tokyo, Japan) equipped with a 16-square reticle. The surface area of this grid was 1 mm^2^. Five separate fields were counted for each filter.

### Ovarian epithelium and carcinoma samples

Between August 2003 and December 2011, normal ovarian tissue, ovarian epithelial benign tumors (serous and mucinous cystadenoma), borderline tumors, primary carcinoma specimens, and metastatic omentum were obtained from patients undergoing surgical resection at the Department of Gynecology, The First Affiliated Hospital of China Medical University (Shenyang, Liaoning, China). The tumor specimens were microscopically confirmed by pathologists. The average age at surgery was 52.1 years (range, 19–80 years). Each ovarian carcinoma specimen was evaluated according to the 2009 FIGO staging system. The histological architecture of ovarian carcinoma was defined in terms of World Health Organization classification. Samples were frozen immediately in liquid nitrogen and stored at −80°C until analysis. No patient had undergone chemotherapy, radiotherapy, or adjuvant treatment before surgery. Informed consent was obtained from all subjects; the China Medical University Ethics Committee approved the study.

### Real-time RT-PCR

Total RNA was extracted from ovarian carcinoma cell lines and ovarian tissues using TRIzol according to the manufacturer’s protocol (Takara, Shiga, Japan). Total RNA (2 μg) was reverse-transcribed to complementary DNA (cDNA) using avian myeloblastosis virus transcriptase and random primers (Takara). The oligonucleotide primers for PCR were based on GenBank sequences. Real-time PCR amplification of the cDNA was performed in 20-μL reactions according to the SYBR Premix Ex Taq™ II kit (Takara); glyceraldehyde-3-phosphate dehydrogenase (GAPDH) was used as the internal control. Hairpin-it™ microRNA and U6 snRNA Normalization RT-PCR Quantitation (GenePharma) was used to check mature miR-93-5P. HmiR-93-Forward: GCC GCC AAA GTG CTG TTC; Reverse: CAG AGC AGG GTC CGA GGTA. U6 was set as the internal control. HU6s-Forward: ATT GGA ACG ATA CAG AGA AGA TT; Reverse: GGA ACG CTT CAC GAA TTT G.

### Western blotting

Cells and tissues were lysed by ice-cold RIPA lysis buffer. The protein concentration was measured by protein assay kit (Bio-Rad Laboratories, Hercules, CA, USA). Subsequently, all denatured protein samples (60 μg each) were separated by 10% SDS-polyacrylamide gel electrophoresis (SDS-PAGE) and then transferred onto Hybond membranes (Amersham, Munich, Germany). Following blocking for 1h in 5% fat-free milk, the membranes were incubated with antibodies against P70S6K, P53, RhoC, Bcl-xL, PARP and MMP9 (Santa Cruz Biotechnology, Santa Cruz, CA, USA). Blots were washed with TBST and then incubated with secondary antibodies (anti-mouse, anti-rabbit, or anti-goat IgG antibodies; 1:1000; Dako, Carpinteria, CA, USA). Bands were visualized on X-ray film (Fujifilm, Tokyo, Japan) using ImageQuant LAS 4000 (Fujifilm) and ECL Plus detection reagents (Santa Cruz Biotechnology). GAPDH (Sigma-Aldrich) was set as the internal control.

### Dual-luciferase reporter assay

HEK293T cells were seeded in 24-well plates with complete medium for 24 h before transfection. RhoC 3′-UTR containing the putative miR-93 binding site or a mutant sequence was designed based on the human RhoC mRNA sequence in GenBank and inserted into the downstream of the firefly luciferase reporter (Promega, Madison, WI, USA). The cultures transciently co-transfected with 50 nM miR-93-5P or mock mimics and 600 ng dual luciferase vector (contain either Wt or mutant 3′-UTR). Luciferase activity was measured by Dual-Luciferase Reporter Assay System after 48 h.

### *In vivo* xenografts

Female BALB/c nude mice [BALB/cASlac-nu, SCXK (Hu) 2012-002), 4–5 weeks old] weighing ~20 g were obtained from Shanghai SLAC Laboratory Animal, Co., Ltd. (Shanghai, China) and housed in a specific pathogen–free environment. Skin tumor xenografts in the nude mice were established by subcutaneous injection of 200 μL PBS containing 5 × 10^6^ exponential-growth OVCAR3 cells with mock or miR-93-5P transfection. All animal manipulations were performed in accordance with the National Institutes of Health Guide for the Care and Use of Laboratory Animals, and were approved by the China Medical University Animal Care and Use Committee.

### Immunohistochemistry

Consecutive tissue sections were deparaffinized with xylene, rehydrated with alcohol, and subjected to antigen retrieval by heating in target retrieval solution (Dako) for 15 min in a microwave oven (Oriental Rotor). The sections were quenched with 3% hydrogen peroxide for 20 min to block endogenous peroxidase activity. Non-specific binding was prevented by adding 5% bovine serum albumin for 5 min. The sections were incubated for 15 min with RhoC antibody, then incubated with HRP-conjugated anti-rabbit antibodies (Dako) for 15 min. All incubations were performed by heating in a microwave oven. After each treatment, the slides were washed three times with TBST for 5 min, and the binding sites were visualized with 3, 3′-diaminobenzidine. After counterstaining with Mayer’s hematoxylin, the sections were dehydrated, cleared, and mounted. Negative controls were prepared by omitting the primary antibody. Optical Density Value was measured.

### Statistical analysis

Statistical analysis was performed using the Spearman correlation test for ranked data and the Mann–Whitney U test and paired samples *t*-test to compare the means of different groups. A p-value of <0.05 was considered statistically significant. SPSS 10.0 (SPSS, Chicago, IL, USA) was used to analyze all data.
